# Stress-Induced Hyperglycemia is a Risk Factor for Surgical-Site Infections in Nondiabetic Patients with Open Leg Fractures

**DOI:** 10.1155/2023/6695648

**Published:** 2023-10-25

**Authors:** Luigi Cianni, Matteo Caredda, Andrea De Fazio, Mattia Basilico, Tommaso Greco, Gianpiero Cazzato, Carlo Perisano, Giulio Maccauro, Raffaele Vitiello

**Affiliations:** ^1^Fondazione Policlinico Universitario A. Gemelli IRCSS, Rome, Italy; ^2^Aurelia Hospital, Rome, Italy

## Abstract

**Background:**

Nondiabetic patients with open leg fractures who have elevated blood glucose levels on arrival in the emergency department have an increased risk of surgical-site infections (SSIs).

**Objective:**

This study evaluates the association between the incidence of SSIs in nondiabetic patients with an open leg fracture and blood glucose levels registered on arrival in the ER. We also analyzed the correlation between patients' days of hospital stay and the incidence of SSIs and the time elapsed between the damage control with external fixation and final fixation and the incidence of SSI.

**Methods:**

We retrospectively studied nondiabetic patients admitted to our emergency unit from 2017 to 2021 with a diagnosis of open leg fracture consecutively treated. Based on the diagnosis of SSIs, all enrolled patients were divided into two groups based on the developed (group A) or not developed (group B) SSIs within 1 year after surgery. All patients enrolled in the study underwent damage control within 24 hours after admission to the ER. At stabilization of general clinical and local wound conditions, all patients underwent definitive surgery.

**Results:**

We enrolled 80 patients. In group A, glycemia on arrival in the ER was on average 148.35 ± 19.59 mg/dl, and in group B, it was 122.61 ± 22.22 mg/dl (*p* value: 0.0001). In group A, glycemia in the first postoperative day was on average 113.81 ± 21.07 mg/dl, and in group B, it was 99.02 ± 17.60 mg/dl (*p* value: 0.001). In group A, the average hospitalization was 57.92 ± 42.43 days, and in group B, it was 18.41 ± 14.21 days (*p* value: 0.01). Through Youden's J, we therefore analyzed the value with the highest sensitivity and specificity which proved to be 132 mg/dl.

**Conclusion:**

Our findings show that nondiabetic patients with SIH have a significantly increased risk of SSIs compared to patients without SIH within 1 year after surgery. Patients with open leg fractures with SIH have a significantly higher average hospital stay than patients without SIH. Further studies are needed to confirm 132 mg/dl of blood glucose levels as a value to stratify the risk of SSIs in these patients.

## 1. Introduction

Open fractures of the lower limb are still difficult injuries to manage for orthopedic surgeons, especially if associated with extensive soft-tissue loss and neurovascular damage [1].

These fractures are relatively uncommon injuries with an estimated incidence of 3.4 open leg fractures per 100,000 people every year and these generally are the consequence of high-energy traumas and most frequently occur in young adult males [2–4].

The primary goal of treatment in open fractures is, beyond the healing of the fracture and restoration of limb function, the prevention of infection [5]. Surgical-site infections (SSIs) are defined by the European Centre for Disease Prevention and Control as microbial contamination of the surgical wound within 30 days of an operation or within 1 year after surgery if an implant is placed in a patient [6].

The incidence of SSIs can vary across surgical procedures, specialties, and conditions, with a reported range of 0.1%–50.4% [7].

SSIs are among the most frequently reported hospital-acquired infections (HAIs), constituting up to 19.6% of all HAIs in Europe in 2011-2012 [8].

Due to prolonged hospitalization, the need for many diagnostic tests, and high reoperation rates, patients who develop an SSI represent a financial burden approximately double to that of patients who do not [9, 10].

Furthermore, longer in-patient stays, loss of earnings during recovery, and increased morbidity as a result of developing an SSI have been related to worse health-related quality of life [11].

For all these reasons, the prevention of SSIs is essential from an economic, social, and patient health point of view [12, 13].

Generous wound irrigation, early surgical debridement within 6 hours, immediate stabilization of the fracture, and antibiotic administration as soon as possible are the most effective measures for preventing infection in the treatment of open fractures [14–16].

Even though these measures are fundamental in order to minimize the risk of infection, other factors must be taken into account.

Among the factors which may contribute to the onset of postoperative infections are trauma energy, size of the lesion, severity of the bone damage, devitalization of soft tissues, and degree of local contamination [17, 18].

The known nonmodifiable patient-related risk factors for developing an infection in open fractures are age, obesity, diabetes mellitus, congestive heart failure, psychiatric illness, bleeding disorders, presence of compartment syndrome, smoking status, and immunodeficiency [19–22].

Despite the many scientific and technological advances, postoperative infection continues to be a large problem in the management of open fractures of the lower limb, with a reported incidence of up to 13% [23, 24]. Another factor reported in the literature which seems to be related with a greater infective risk in trauma patients is hyperglycemia. Stress-induced hyperglycemia (SIH) is not a new concept considering that almost a third of ill patients admitted to the hospital with no history of diabetes have hyperglycemia [25].

A normal physiological response to injury can lead to impaired endogenous hormone production and metabolites, including increased serum cortisol and glucagon production, temporary insulin resistance and deficiency, and subsequent hyperglycemia [26, 27].

Several investigations suggest that a stress-induced hyperglycemic response after significant trauma is strongly associated with a longer hospital stay, higher admission rates to the intensive care unit, increased risk of infections, and mortality [28–30].

Although significant contributions concerning the topic of perioperative hyperglycemia and outcomes have been made to the general surgery and critical care literature [32–35], this topic remains quite overlooked in orthopedic surgery. In fact, relatively few studies can be found in the orthopedic literature which correlates hyperglycemia with an increased risk of infection [31, 36–40] and, to our knowledge, none specifically concern nondiabetic patients with open leg fractures.

The aim of our study is to evaluate the correlation between posttraumatic stress-induced hyperglycemia and surgical-site infections in nondiabetic patients undergoing surgery for open leg fractures.

## 2. Materials and Methods

### 2.1. Study Setting and Design

The present investigation is a retrospective observational analysis performed according to the Strengthening the Reporting of Observational Studies in Epidemiology (STROBE) guidelines. Clinical records of nondiabetic patients admitted to our emergency room (ER) from 1^st^ January 2017 to 31^st^ December 2021 with a diagnosis of open leg fracture consecutively treated were potentially eligible for this study. This study was conducted in accordance with the ethical standards of the institutional and national research committee and with the 1964 Helsinki Declaration and its later amendments or comparable ethical standards. A written informed consent for scientific purposes and clinical data collection was obtained according to the institutional protocol.

### 2.2. Inclusion and Exclusion Criteria

Inclusion criteria were as follows: (I) age>18 years, (II) diagnosis of open leg fracture requiring surgical treatment managed in our institution according to Gustilo and Anderson's classification, (III) all patients who were treated at first with external fixation (EF), (IV) nondiabetic patients according to American Diabetes Association criteria: fasting plasma glucose (FPG) ≥126 mg/dL (two findings) or random plasma glucose ≥200 mg/dL or [41], (V) at least a one-year follow-up, and (VI) patients fasting for at least two hours on admission to the emergency department.

Exclusion criteria were as follows: (I) patients with a history of diabetes or diagnosis that occurred during hospitalization, (II) incomplete dataset, (III) use of drugs that affect blood glucose (corticosteroid, etc.), and (IV) patients who have had plastic surgery (V).

### 2.3. Definition of Surgical-Site Infection

SSIs are defined by the ECDC as microbial contamination of the surgical wound within 30 days of an operation or within 1 year after surgery if an implant is placed in a patient [6]. The incidence of SSIs can vary across surgical procedures, specialties, and conditions, with a reported range of 0.1%–50.4% [7]. SSIs are among the most frequently reported HAIs constituting up to 19.6% of all HAIs in Europe in 2011-2012 [8].

### 2.4. Outcomes

The primary outcome was the association between the incidence of SSI in nondiabetic patients with an open leg fracture and blood glucose levels registered on arrival in the ER. The secondary outcomes were the correlation between patients' days of hospital stay and the incidence of SSI and the time elapsed between the damage control with final fixation and the incidence of SSI.

### 2.5. Institutional Database and Data Collection

All nondiabetic patients with a diagnosis of open leg fracture treated in the emergency unit (EU) of our institution were managed using a standardized data collection system. From each patient, the following data were collected and stored: age, gender, clinical history, body mass index (BMI), routine blood tests, type of fracture, type of surgery performed, period between damage control in the EU and definitive surgical treatment, and incidence of SSI within 1 year after surgery.

### 2.6. Patients's Management and Group Settings

Based on the diagnosis of SSIs, all enrolled patients were divided into two groups. In group A, we included patients with SSIs, while in group B we collocated patients without SSIs. All patients enrolled in the study underwent EF surgery and accurate debridement within 24 hours after admission to the ER. All patients received antibiotic therapy for infection prevention according to the type of trauma and internal guidelines [42]. All blood tests were performed at ER arrival, on the first and third day after surgery and then based on the surgeon's assessment. The surgical indication was established by experienced orthopedic and trauma surgeons according to the latest guidelines. Generally, the same surgical technique was performed to treat the same type of fracture [42]. At stabilization of general clinical and local wound conditions, all patients underwent definitive surgery. In this case, patients underwent two different types of surgery: intramedullary nailing or osteosynthesis with plates and screws. Starting with the first postoperative day, all patients followed a physiotherapy protocol including, when possible, partial, and then progressive load according to the type of fracture and surgeon's indications. As the standard of care in our institution, all patients with a diagnosis of open leg fracture surgically treated were systematically monitored at 1, 3, 6, and 12 months after surgery. The complication rate was recorded during the follow-up period.

### 2.7. Statistical Analysis

Statistical Analysis GraphPad QuickCalcs (GraphPad Software, San Diego, CA, USA) was used for data analysis. The data were reported as mean and standard deviation (+SD). The asymmetry was calculated to evaluate the normality of the different parameters. An unpaired *t*-test was used to compare unrelated, continuous, variables. A multiple regression was performed on more incisive variables. ROC analysis and Youden's J test were used to analyze the sensitivity and sensibility of stress-induced hyperglycemia to predict SSI and best its cut-off. Significance was set for *p* < 0.05.

## 3. Results

In our study, we recruited 80 patients, of which 20 were females and 60 were males.

The average age of patients was 45.87 ± 17.21. The average BMI of patients was 24.57 ± 3.09.

Twenty patients reported dislocation with open fracture and sixty patients reported only open fractures.

All patients were treated with damage control. All patients were treated in the EU with surgical debridement, irrigation, and positioning of monoplanar EF within 24 hours after admission to the ER (Table 1).

43 patients were treated with plate and screws and 37 patients were treated with intramedullary nails.

We divided the 80 patients recruited into 2 groups. A group A of patients who developed SSIs within 1 year after surgery and another group B of patients who did not develop SSIs within 1 year after surgery.

In group A, 23 patients were male and 3 patients were female. In group B, 37 patients were male and 17 were female.

The average age of patients in group A was 47.46 ± 17.34 and in group B was 45.50 ± 17.36.

The average BMI of patients in group A was 24.33 ± 2.64 and in group B was 24.65 ± 3.44.

In group A, 8 patients reported a dislocation with open fracture, while in group B, 12 patients reported a dislocation with open fracture.

In group A 14 patients were treated with plate and screws and 12 patients were treated with intramedullary nails. In group B 23 patients were treated with plate and screws and 20 patients were treated with intramedullary nails.

In group A, the average hospitalization was 57.92 ± 42.43 days, and in group B it was 18.41 ± 14.21 days (*p* value: 0.01) (Table 2).

The days elapsed between the EF and synthesis in groups A and B were 29.00 ± 35.50 and 20.78 ± 30.54 (*p* value: 0.02) (Table 2).

In group A, glycemia on arrival in the ER was on average 148.35 ± 19.59 mg/dl, and in group B, it was 122.61 ± 22.22 mg/dl (*p* value 0.0001) (Table 3).

In group A, glycemia in the first postoperative day was on average 113.81 ± 21.07 mg/dl, and in group B, it was 99.02 ± 17.60 mg/dl (*p* value: 0.001) (Table 3).

In group A, glycemia in the third postoperative day was on average 100.13 ± 18.84 mg/dl, and in group B, it was 95.07 ± 10.00 mg/dl (*p* value: 0.2) (Table 3).

In group A, bacteria were isolated from 18 patients, wherein in 5 patients isolations were monomicrobial, while in 13 patients isolations were polymicrobial. The most frequently isolated bacteria were in 8 patients Enterobacteriaceae (mainly *E. coli, Ent. faecalis,* and *Ent. cloacae*), in 6 patients *Pseudomonas aeruginosa*, in 5 patients Acinetobacter, and in 4 patients *St. aureus* (of which 2 is MRSA).

In group A, SSIs occurred in 17 patients before final synthesis and in 9 patients after final synthesis.

The association between the choice of synthesis type and 'onset of SSI was not found to be statistically significant in both cases (*p* value 0.2−0.1).

**Figure 1:**
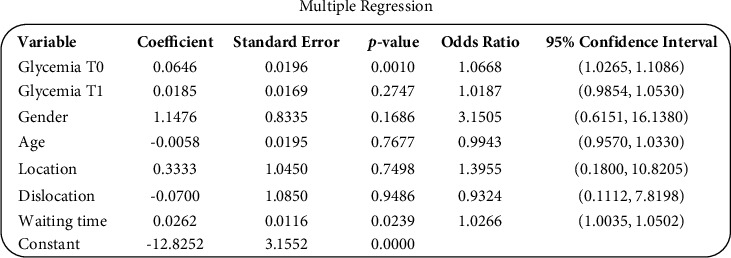
Patients' clinical and surgical variables.

The parameters were then used to perform a multiple regression (Figure 1). The regression showed that the only the significant parameter remaining is the blood glucose upon arrival in the ER (*p*=0.001; OR: 1.06) and the waiting days between damage control and definitive synthesis (*p*=0.02; OR: 1.02).

**Figure 2:**
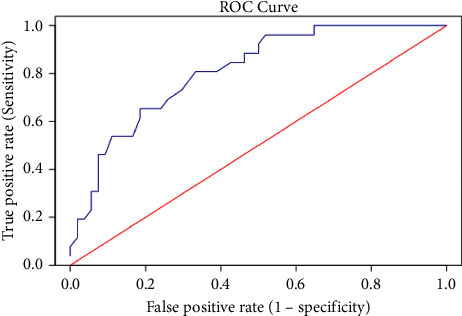
Sensitivity and specificity of blood glucose value upon arrival in the ER for predicting SSIs.

**Figure 3:**
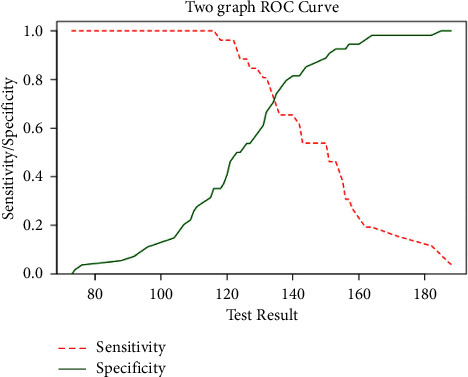
Identification of the glucose value with the highest sensitivity and specificity through Youden's J.

In light of this result, we studied the sensitivity and specificity of blood glucose upon arrival in the ER in predicting SSIs. The variable demonstrated a good AUC of 0.81 (95% CI: 0.71–0.9) (Figure 2). Through Youden's J, we therefore analyzed the value with the highest sensitivity and specificity which proved to be 132 mg/dl, demonstrating a sensitivity of 0.80 and a specificity of 0.67 (Figure 3).

## 4. Discussion

Open leg fractures are intrinsically high-infectious-risk injuries due to soft-tissue damage and local contamination [12]. Surgical-site infections (SSIs) are unlucky occurrences after orthopedic trauma surgery, and their consequences represent a considerable burden for both patients and the healthcare system [11].

Several factors related to an increased risk of SSIs have been reported in the literature [39–41]. Our study correlates the posttraumatic hyperglycemia recorded in ER in nondiabetic patients with open leg fractures to an increased risk of SSIs. The correlation between complications such as SSI and diabetes is discussed and it is unique in the literature. Shao et al. in a 2018 review showed that diabetes is a risk factor for SSI in patients undergoing surgery after ankle fractures [43].

Schmidt et al. in 2020 investigated the effects of diabetes on ankle fractures. The authors analyzed 979 patients, of which 131 were diabetic patients. Diabetic patients had a complication rate of 26%, that is, a higher incidence than nondiabetic patients [44]. In our study, we did not consider diabetic patients. In the literature, we described a 25% rate of infection in diabetic patients with exposed leg fractures versus 13% in nondiabetic patients [45]. In our work, we noticed that the incidence values of SSIs are higher than the data in the literature. The hyperglycemic patients we recruited have a doubled infectious risk compared to nonhyperglycemic patients, considering a hyperglycemic patient with a value > 132 mg/dl as reported in our ROC curve at PS entry. Thus, patients with SIH demonstrate an increased infectious risk as diabetic patients.

The relationship between hyperglycemia and infection is not a new concept in literature. Stress-induced hyperglycemia (SIH) is a physiological phenomenon which may occur in both diabetic and nondiabetic patients following a significant trauma.

Diabetic hyperglycemia (DH) is certainly the most studied in the literature, however, several articles reported that SIH, and not DH, is mainly responsible for the higher rate of morbidity and mortality in trauma patients [46–48]. SIH is an acute process that is known to interfere with monocyte and neutrophil function, which increases the risk of infection [49]. The perioperative period is often associated with poor oral intake in the setting of increased metabolic demand. Our cells respond by resorting to gluconeogenesis from amino acids and glycerol glycogenolysis. Due to these metabolic changes, patients with reduced glucose tolerance may incur into hyperglycemia [50].

In a recent study, Rau et al. analyzed the differential impact of hyperglycemia on outcomes between diabetic and nondiabetic trauma inpatients and they reported that nondiabetic patients with hyperglycemia had higher mortality and worse outcomes than the diabetic patients [47].

Similarly, Mamtani et al. reported a higher morbidity and mortality among trauma patients with SIH than with DH [48].

The secondary outcome of our study is the correlation between SIH and an increased length of hospital stay. We show that patients with open leg fractures and SIH have longer hospitalization than normoglycemic patients.

According to our findings, Delamaire et al. reported that hyperglycemia in nondiabetic critically ill patients is associated with longer hospitalizations and higher in-hospital mortality, especially in patients with trauma, neurological disease, and coma patients [49].

The association between hyperglycemia and the onset of SSIs has also been reported in the orthopedic literature [39–41]. Most of the studies concern the correlation between postoperative hyperglycemia and the onset of infections, and not the admission hyperglycemia like in our study. In addition, we have specifically reported the correlation between SIH and SSIs only in nondiabetic patients with open leg fractures, which, to our knowledge, has never been reported before in the literature. Furthermore, relatively few studies have evaluated the relationship between hyperglycemia and SSIs in a nondiabetic population.

Richards et collegues in a study including only nondiabetic patients with isolated orthopedic injuries requiring acute operative intervention reported that postoperative hyperglycemia is strongly associated with 30-day SSIs; however, admission glucose was not associated with subsequent SSI (124.6 (27.0) mg/dL vs. 116.9 (23.5) mg/dL, *p*=0.28), contrarily to our study [41]. Furthermore, this study considers patients with generic orthopedic trauma and not specifically patients with open leg fractures.

Richards et al. reported that in nondiabetic patients, a morning blood glucose greater than 140 mg/dL on postoperative day 1 had a threefold increase in the risk for infection compared to normoglycemic patients [38]. However, this study considers the postoperative glycemia and not the admission one and concerns elective surgery.

The ability to predict which patients are at risk for development of SSI is of clinical interest because it could be a first step in preventing infection and it could result in more effective therapeutic measures for each patient [51, 52].

Our study demonstrates the correlation between SIH and the onset of SSIs in patients with open leg fractures. As secondary outcomes, we have demonstrated the association between the onset of SSIs and longer hospitalization and longer time elapsed between damage control and definitive surgical treatment and onset of SSIs.

In our study we have also defined, through Youden's J, the cut-off value of blood glucose above which the risk of SSIs is higher, thus demonstrating a sensitivity of 0.80 and a specificity of 0.67.

Thus, in patients with a blood glucose value greater than 132 mg/dL in ER and by considering the increased risk of SSIs, more effective protective strategies should be implemented.

The number of the population taken into consideration is quite small. It should be noted, however, that it is a court of patients selected with selective inclusion and exclusion criteria. We recruited only nondiabetic patients with open leg fractures, who underwent a follow-up period of at least one year and who had been fasting for at least two hours before admission to the ER.

This is a preliminary study but with significant statistical evidence. Further large prospective randomized studies are needed to better describe the correlation between hyperglycemia and the onset of SSIs in nondiabetic patients with open leg fractures. A better comprehension of these mechanisms could result in more effective treatments for these delicate patients.

## 5. Conclusions

Open leg fractures are still difficult injuries to manage for orthopedic surgeons. One of the most adverse and frequent complications in these patients are the SSIs. The literature has already shown a correlation between SSIs and SIH but no author had done so for nondiabetic patients with open leg fractures. Our preliminary findings show that nondiabetic patients with a blood glucose value recorded in ER ≥ 132 mg/dL have a significantly increased risk of SSIs compared to patients without SIH within 1 year after surgery. Thus, in these patients, more effective protective strategies should be implemented in order to prevent the onset of SSIs. Patients with open leg fractures with SIH have a significantly higher average hospital stay than patients without SIH. Further studies with larger populations will be necessary to deepen this issue and to ensure a correct stratification of the risk of these patients on arrival in the ER and may consider a possible benefit of medical therapy in SIH patients with exposed leg fractures. In addition, other studies are needed to confirm that 132 mg/dl of blood glucose level is a reliable cut-off value to stratify the risk of SSIs in these patients.

## Figures and Tables

**Table 1:** Patients' clinical and surgical characteristics.

	Group A	Group B
Number of patients	26	54
Gender	23 M	37 M
	3 F	17 F
Average age	47.46 ± 17.34	45.50 ± 17.36
BMI	24.33 ± 2.64	24.65 ± 3.44
Number of dislocation	8	12
Damage control and external fixation	26 (all patients)	54 (all patients)
Type of synthesis	20 plates and screws	23 plates and screws
	17 nail	20 nail

M = male; F = female; BMI = body mass index.

**Table 2:** Surgical information.

	Group A	Group B	*P* value
Average stay	57.92 ± 42.43	18.41 ± 14.21	0.01
Days between EF and synthesis	29.00 ± 35.50	20.78 ± 30.54	0.02

EF = external fixator.

**Table 3:** Patients' glycemia.

	Group A	Group B	*P* value
Glycemia in ER	148.35 ± 19.59	122.61 ± 22.22	0.0001
Glycemia in POD 1	113.81 ± 21.07	99.02 ± 17.60	0.001
Glycemia in POD 3	100.13 ± 18.84	95.07 ± 10.00	0.2

ER = emergency room; POD = postoperative day.

## Data Availability

The datasets generated and/or analysed during the current study are available from the corresponding author on reasonable request.

## References

[1] Gustilo R. B., Anderson J. T. (1976). Prevention of infection in the treatment of one thousand and twenty-five open fractures of long bones: retrospective and prospective analyses. *The Journal of Bone and Joint Surgery*.

[2] Court-Brown C. M., Bugler K. E., Clement N. D., Duckworth A. D., McQueen M. M. (2012). The epidemiology of open fractures in adults. A 15-year review. *Injury*.

[3] Larsen P., Elsoe R., Hansen S. H., Graven-Nielsen T., Laessoe U., Rasmussen S. (2015). Incidence and epidemiology of tibial shaft fractures. *Injury*.

[4] Court-Brown C. M., Rimmer S., Prakash U., McQueen M. M. (1998). The epidemiology of open long bone fractures. *Injury*.

[5] Zalavras C. G. (2017). Prevention of infection in open fractures. *Infectious Disease Clinics of North America*.

[6] Horan T. C., Gaynes R. P., Martone W. J., Jarvis W. R., Graceemori T. (1992). CDC definitions of nosocomial surgical site infections, 1992: a modification of CDC definitions of surgical wound infections. *American Journal of Infection Control*.

[7] Korol E., Johnston K., Waser N. (2013). A systematic review of risk factors associated with surgical site infections among surgical patients. *PLoS One*.

[8] Suetens C., Latour K., Kärki T. (2018). Prevalence of healthcare-associated infections, estimated incidence and composite antimicrobial resistance index in acute care hospitals and long-term care facilities: results from two European point prevalence surveys, 2016 to 2017. *Euro Surveillance*.

[9] Broex E. C. J., van Asselt A. D. I., Bruggeman C. A., van Tiel F. H. (2009). Surgical site infections: how high are the costs?. *Journal of Hospital Infection*.

[10] Parker B., Petrou S., Masters J. P. M., Achana F., Costa M. L. (2018). Economic outcomes associated with deep surgical site infection in patients with an open fracture of the lower limb. *The Bone and Joint Journal*.

[11] Pinkney T. D., Calvert M., Bartlett D. C. (2013). Impact of wound edge protection devices on surgical site infection after laparotomy: multicentre randomised controlled trial (ROSSINI Trial). *British medical journal*.

[12] Basilico M., Vitiello R., Oliva M. S. (2020). Predictable risk factors for infections in proximal femur fractures. *Journal of Biological Regulators and Homeostatic Agents*.

[13] Greco T., Vitiello R., Cazzato G. (2020). Intramedullary antibiotic coated nail in tibial fracture: a systematic review. *Journal of Biological Regulators & Homeostatic Agents*.

[14] Bhandari M., Jeray K. J., Petrisor B. A. (2015). A trial of wound irrigation in the initial management of open fracture wounds. *New England Journal of Medicine*.

[15] Prodromidis A. D., Charalambous C. P. (2016). The 6-hour rule for surgical debridement of open tibial fractures: a systematic review and meta-analysis of infection and nonunion rates. *Journal of Orthopaedic Trauma*.

[16] Rupp M., Popp D., Alt V. (2020). Prevention of infection in open fractures: where are the pendulums now? Injury. *Injury*.

[17] Yusof N. M., Khalid K. A., Zulkifly A. H. (2013). Factors associated with the outcome of open tibial fractures. *Malaysian Journal of Medical Sciences: MJMS*.

[18] Hull P. D., Johnson S. C., Stephen D. J. G., Kreder H. J., Jenkinson R. J. (2014). Delayed debridement of severe open fractures is associated with a higher rate of deep infection. *The Bone and Joint Journal*.

[19] Khatod M., Botte M. J., Hoyt D. B., Meyer R. S., Smith J. M., Akeson W. H. (2003). Outcomes in open tibia fractures: relationship between delay in treatment and infection. *The Journal of Trauma, Injury, Infection, and Critical Care*.

[20] Castillo R. C., Bosse M. J., MacKenzie E. J., Patterson B. M. (2005). Impact of smoking on fracture healing and risk of complications in limb-threatening open tibia fractures. *Journal of Orthopaedic Trauma*.

[21] Saiz A. M., Stwalley D., Wolinsky P., Miller A. N. (2022). Patient comorbidities associated with acute infection after open tibial fractures. *Journal of the American Academy of Orthopaedic Surgeons. Global research & reviews*.

[22] Willy C., Rieger H., Stichling M. (2017). Prevention of postoperative infections: risk factors and the current WHO guidelines in musculoskeletal surgery. *Unfallchirurg, Der*.

[23] Leonidou A., Kiraly Z., Gality H., Apperley S., Vanstone S., Woods D. A. (2014). The effect of the timing of antibiotics and surgical treatment on infection rates in open long-bone fractures: a 6-year prospective study after a change in policy. *Strategies in Trauma and Limb Reconstruction*.

[24] Youbong T. J., De Pontfarcy A., Rouyer M. (2021). Bacterial epidemiology of surgical site infections after open fractures of the lower limb: a retrospective cohort study. *Antibiotics*.

[25] Levetan C. S., Passaro M., Jablonski K., Kass M., Ratner R. E. (1998). Unrecognized diabetes among hospitalized patients. *Diabetes Care*.

[26] Ali Abdelhamid Y., Kar P., Finnis M. E. (2016). Stress hyperglycaemia in critically ill patients and the subsequent risk of diabetes: a systematic review and meta-analysis. *Critical Care*.

[27] Vedantam D., Poman D. S., Motwani L., Asif N., Patel A., Anne K. K. (2022). Stress-induced hyperglycemia: consequences and management. *Cureus*.

[28] Bochicchio G. V., Salzano L., Joshi M., Bochicchio K., Scalea T. M. (2005). Admission preoperative glucose is predictive of morbidity and mortality in trauma patients who require immediate operative intervention. *The American Surgeon*.

[29] Sung J., Bochicchio G. V., Joshi M., Bochicchio K., Tracy K., Scalea T. M. (2005). Admission hyperglycemia is predictive of outcome in critically ill trauma patients. *The Journal of Trauma, Injury, Infection, and Critical Care*.

[30] Karunakar M. A., Staples K. S. (2010). Does stress-induced hyperglycemia increase the risk of perioperative infectious complications in orthopaedic trauma patients?. *Journal of Orthopaedic Trauma*.

[31] Ata A., Lee J., Bestle S. L., Desemone J., Stain S. C. (2010). Postoperative hyperglycemia and surgical site infection in general surgery patients. *Archives of Surgery*.

[32] Chang M. W., Huang C. Y., Liu H. T., Chen Y. C., Hsieh C. H. (2018). Stress-induced and diabetic hyperglycemia associated with higher mortality among intensive care unit trauma patients: cross-sectional analysis of the propensity score-matched population. *International Journal of Environmental Research and Public Health*.

[33] Finfer S., Chittock D. R., Su S. Y. S. (2009). Intensive versus conventional glucose control in critically ill patients. *New England Journal of Medicine*.

[34] Bochicchio G. V., Bochicchio K. M., Joshi M., Ilahi O., Scalea T. M. (2010). Acute glucose elevation is highly predictive of infection and outcome in critically injured trauma patients. *Annals of Surgery*.

[35] Ramos M., Khalpey Z., Lipsitz S. (2008). Relationship of perioperative hyperglycemia and postoperative infections in patients who undergo general and vascular surgery. *Annals of Surgery*.

[36] Rizvi A. A., Chillag S. A., Chillag K. J. (2010). Perioperative management of diabetes and hyperglycemia in patients undergoing orthopaedic surgery. *American Academy of Orthopaedic Surgeon*.

[37] Mraovic B., Suh D., Jacovides C., Parvizi J. (2011). Perioperative hyperglycemia and postoperative infection after lower limb arthroplasty. *Journal of Diabetes Science and Technology*.

[38] Richards J. E., Kauffmann R. M., Zuckerman S. L., Obremskey W. T., May A. K. (2012). Relationship of hyperglycemia and surgical-site infection in orthopaedic surgery. *The Journal of Bone and Joint Surgery*.

[39] Richards J. E., Kauffmann R. M., Obremskey W. T., May A. K. (2013). Stress-induced hyperglycemia as a risk factor for surgical-site infection in nondiabetic orthopedic trauma patients admitted to the intensive care unit. *Journal of Orthopaedic Trauma*.

[40] Richards J. E., Hutchinson J., Mukherjee K. (2014). Stress hyperglycemia and surgical site infection in stable nondiabetic adults with orthopedic injuries. *Journal of Trauma and Acute Care Surgery*.

[41] American Diabetes Association (2021). 2. Classification and diagnosis of diabetes: standards of medical care in diabetes-2021. *Diabetes Care*.

[42] Rymer B., Dimovska E. O. F., Chou D. T. S., Choa R., Davis B., Huq S. (2017). A representative assessment of the management of open fractures of the lower limb within UK orthoplastic centres: a two-centre audit of compliance with national standards. *Injury*.

[43] Shao J., Zhang H., Yin B., Li J., Zhu Y., Zhang Y. (2018). Risk factors for surgical site infection following operative treatment of ankle fractures: a systematic review and meta-analysis. *International Journal of Surgery*.

[44] Schmidt T., Simske N. M., Audet M. A., Benedick A., Kim C. Y., Vallier H. A. (2020). Effects of diabetes mellitus on functional outcomes and complications after torsional ankle fracture. *Journal of the American Academy of Orthopaedic Surgeons*.

[45] Kortram K., Bezstarosti H., Metsemakers W. J., Raschke M. J., Van Lieshout E. M. M., Verhofstad M. H. J. (2017). Risk factors for infectious complications after open fractures; a systematic review and meta-analysis. *International Orthopaedics*.

[46] Fakhry S. M., Morse J. L., Wilson N. Y. (2022). Hyperglycemia in nondiabetic adult trauma patients is associated with worse outcomes than diabetic patients: an analysis of 95,764 patients. *Journal of Trauma and Acute Care Surgery*.

[47] Rau C. S., Wu S. C., Chen Y. C. (2017). Higher mortality in trauma patients is associated with stress-induced hyperglycemia, but not diabetic hyperglycemia: a cross-sectional analysis based on a propensity-score matching approach. *International Journal of Environmental Research and Public Health*.

[48] Mamtani M., Kulkarni H., Bihari S. (2020). Degree of hyperglycemia independently associates with hospital mortality and length of stay in critically ill, nondiabetic patients: results from the ANZICS CORE binational registry. *Journal of Critical Care*.

[49] Delamaire M., Maugendre D., Moreno M., Le Goff M. C., Allannic H., Genetet B. (1997). Impaired leucocyte functions in diabetic patients. *Diabetic Medicine*.

[50] Lee P., Min L., Mody L. (2014). Perioperative glucose control and infection risk in older surgical patients. *Current Geriatrics Reports*.

[51] van Walraven C., Musselman R. (2013). The surgical site infection risk score (ssirs): a model to predict the risk of surgical site infections. *PLoS One*.

[52] Matos M. A., Lima L. G., de Oliveira L. A. A. (2015). Predisposing factors for early infection in patients with open fractures and proposal for a risk score. *Journal of Orthopaedics and Traumatology*.

